# Supplementary material for: Cinnamides Target Leishmania amazonensis Arginase Selectively
Source: Molecules. 2020 Nov 12;25(22):5271. doi: 10.3390/molecules25225271 (PMC7696938; doi:10.3390/molecules25225271)
Supplement: Supplementary file 1 [file molecules-25-05271-s001.pdf]

Supplementary Information

Article

Cinnamides target *Leishmania amazonensis* arginase selectively

Edson Roberto da Silva [1,*], Júlio Abel Alfredo dos Santos Simone Come [1,2], Simone Brogi [3,*], Vincenzo Calderone [3], Giulia Chemi [4,¥], Giuseppe Campiani [4], Trícia Maria Ferrreira de Sousa Oliveira [1], Thanh-Nhat Pham [5], Marc Pudlo [5], Corine Girard [5,*], and Claudia do Carmo Maquiaveli [1,*]

[1] Universidade de São Paulo Faculdade de Zootecnia e Engenharia de Alimentos, Departamento de Medicina Veterinária, Laboratório de Farmacologia e Bioquímica (LFBq), Pirassununga, SP, 13635-900, Brasil.
[2] Universidade Eduardo Mondlane, Faculdade de Veterinária, Departamento de Pré-Clínicas, Av. de Moçambique, Km 1.5, CP 257, Maputo, Moçambique.
[3] Department of Pharmacy, University of Pisa, via Bonanno 6, 56126 Pisa, Italy
[4] Department of Biotechnology, Chemistry, and Pharmacy, DoE Department of Excellence 2018-2022 Università degli Studi di Siena via Aldo Moro 2, 53100, Siena, Italy
[5] University Bourgogne Franche-Comté, PEPITE EA4267, F-25000 Besançon, France
¥Present address: Wellcome Centre for Anti-Infectives Research, Drug Discovery Unit, Division of Biological Chemistry and Drug Discovery, University of Dundee, DD1 5EH Dundee, United Kingdom

* Correspondence: edsilva@usp.br (ES), cmaquiaveli40@gmail.com (CM), simone.brogi@unipi.it (SB), corine.girard@univ-fcomte.fr (CG)

Table of Contents

Figure S1 **page S2**
Figure S2 **page S2**
Figure S3 **page S3**
Figure S4 **page S4**
Figure S5 **page S5**
Figure S6 **page S6**
Figure S7 **page S7**

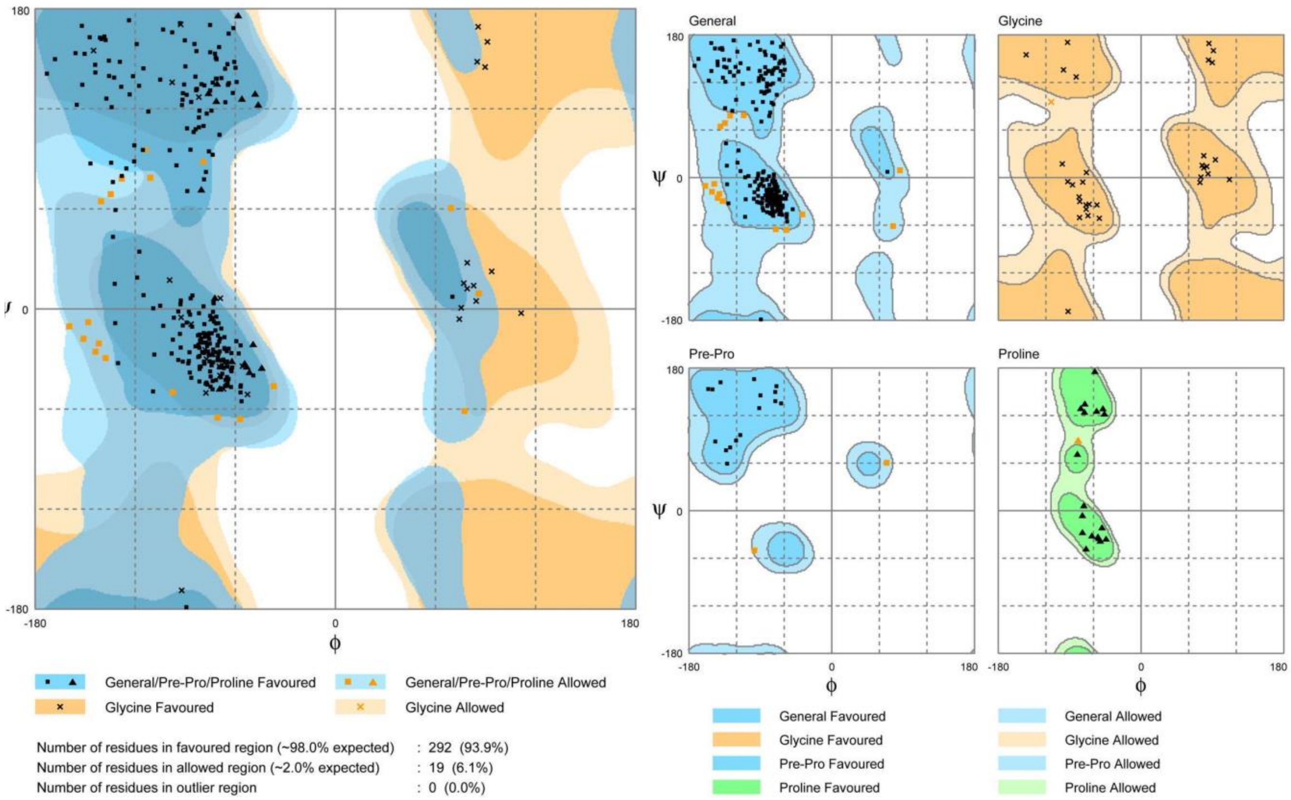

Figure S1. Ramachandran plot analysis of the modeled bovine arginase. (Right): general panel; (Left): representation of the placement of residues in homology modeled bovine arginase for general Gly, Pre-Pro, and Pro, obtained by means of RAMPAGE web server (http://mordred.bioc.cam.ac.uk/~rapper/rampage.php).

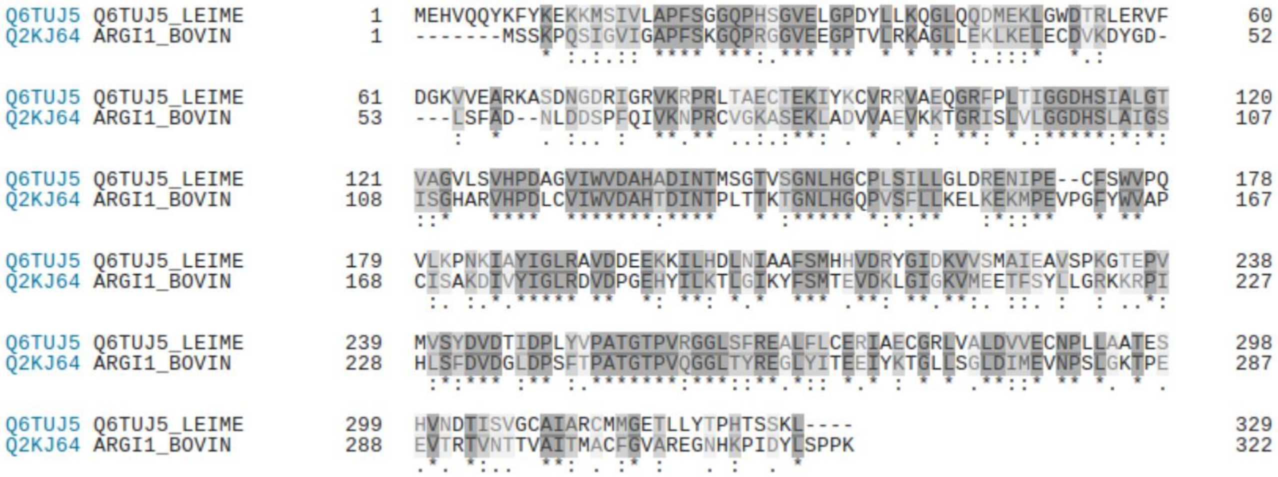

Figure S2. *L. mexicana arginase* and bovine arginase sequence alignment as performed by means of the Clustal Omega program accessible via the UniprotKB web server (http://www.uniprot.org/align).
*Consensus sequence

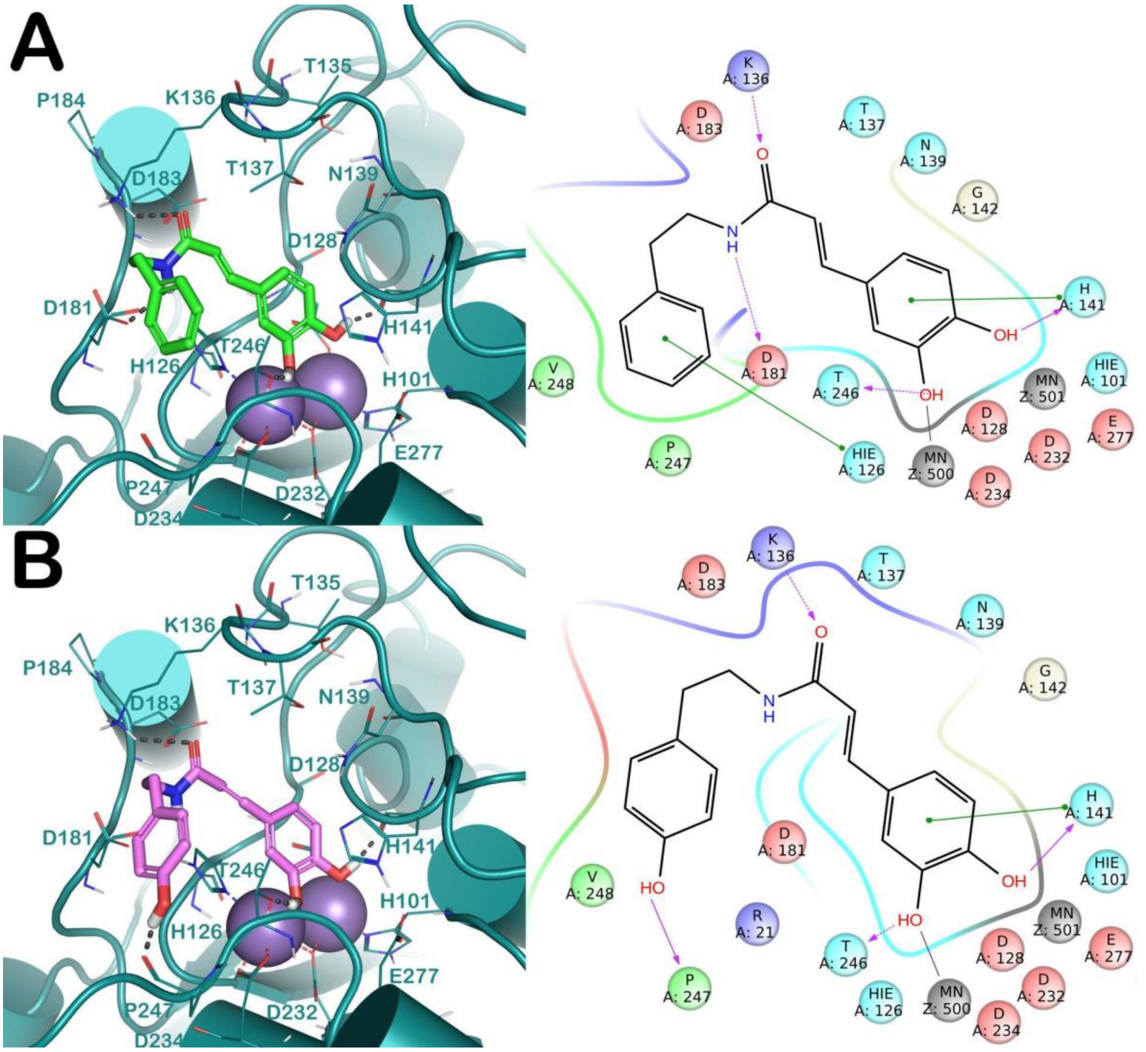

Figure S3. (A) Putative binding mode of CAPA (green sticks) into bovine arginase binding site (deep green cartoon) as found by Glide software. Metals are represented as gray spheres. Key residues of the binding site are represented by lines. H-bonds are represented as black dotted lines, while the metal coordination bonds are represented by colored dotted lines. On the right panel is reported the ligand interaction diagram. (B) Putative binding mode of compound **6** (pink sticks) into bovine arginase binding site (deep green cartoon) as found by Glide software. Metals are represented as gray spheres. Key residues of the binding site are represented by lines. H-bonds are represented as black dotted lines, while the metal coordination bonds are represented by colored dotted lines. On the right panel is reported the ligand interaction diagram The picture was generated by means of PyMOL (The PyMOL Molecular Graphics System, v1.8; Schrodinger, LLC, New York, 2015), while the ligand interaction diagram was generated by Maestro (Maestro, Schrödinger, LLC, New York, NY, 2016).

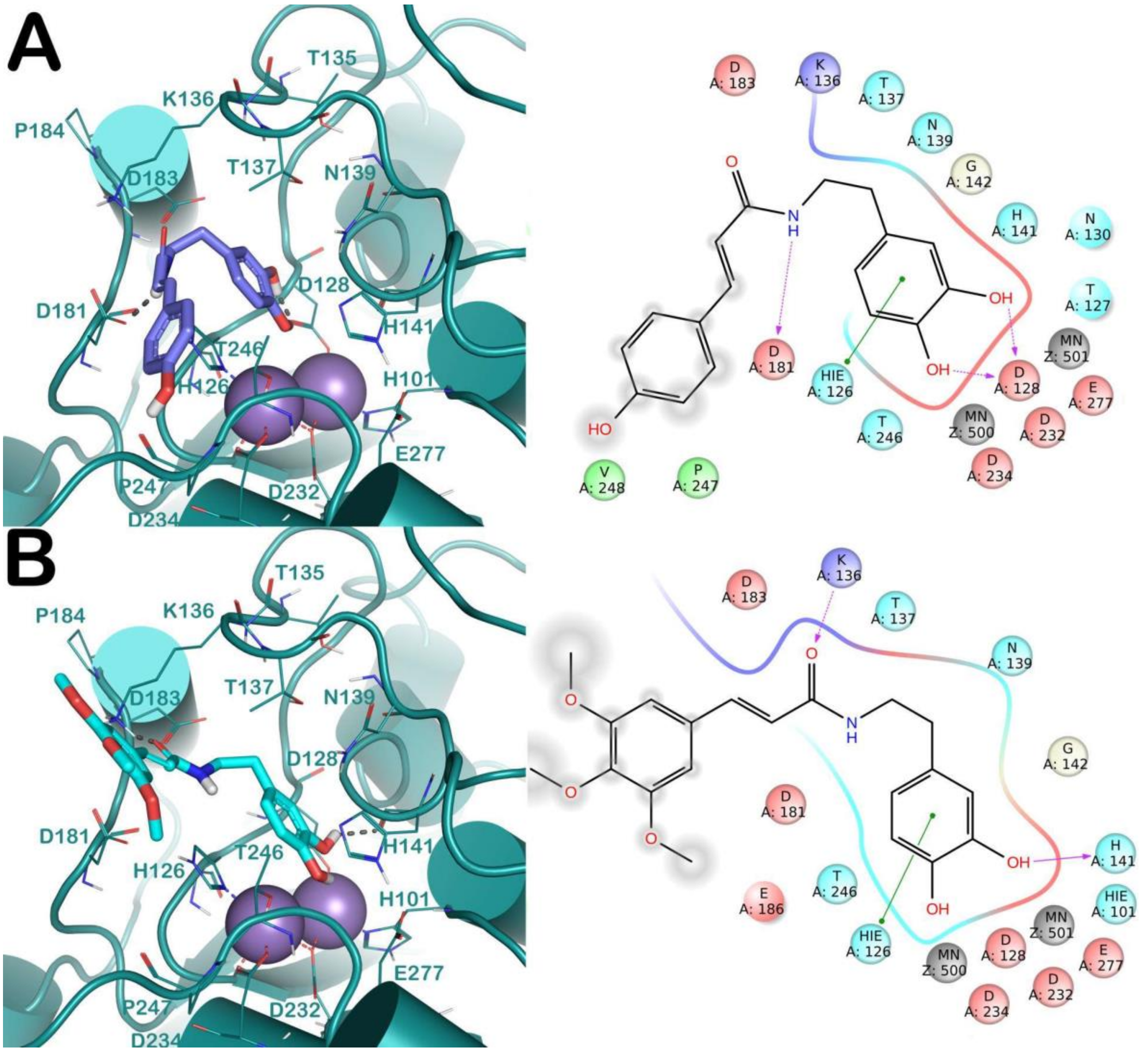

Figure S4. (A) Putative binding mode of compound **13** (blue sticks) into bovine arginase binding site (deep green cartoon) as found by Glide software. Metals are represented as gray spheres. Key residues of the binding site are represented by lines. H-bonds are represented as black dotted lines, while the metal coordination bonds are represented by colored dotted lines. On the right panel is reported the ligand interaction diagram. (B) Putative binding mode of compound **14** (cyan sticks) into bovine arginase binding site (deep green cartoon) as found by Glide software. Metals are represented as gray spheres. Key residues of the binding site are represented by lines. H-bonds are represented as black dotted lines, while the metal coordination bonds are represented by colored dotted lines. On the right panel is reported the ligand interaction diagram The picture was generated by means of PyMOL (The PyMOL Molecular Graphics System, v1.8; Schrodinger, LLC, New York, 2015), while the ligand interaction diagram was generated by Maestro (Maestro, Schrödinger, LLC, New York, NY, 2016).

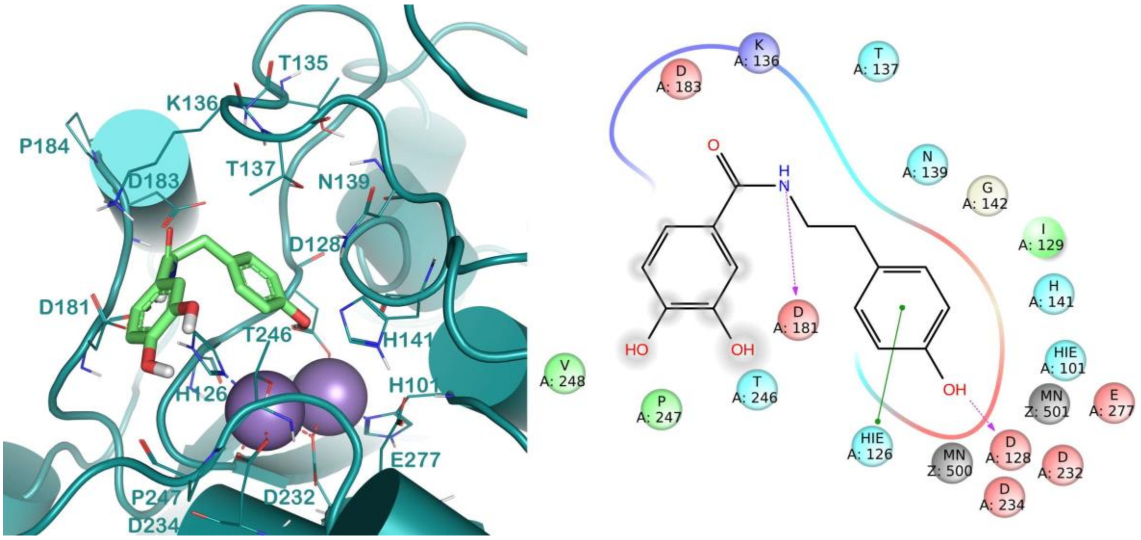

Figure S5. Putative binding mode of compound **17** (light green sticks) into bovine arginase binding site (deep green cartoon) as found by Glide software. Metals are represented as gray spheres. Key residues of the binding site are represented by lines. H-bonds are represented as black dotted lines, while the metal coordination bonds are represented by colored dotted lines. On the right panel is reported the ligand interaction diagram The picture was generated by means of PyMOL (The PyMOL Molecular Graphics System, v1.8; Schrodinger, LLC, New York, 2015), while the ligand interaction diagram was generated by Maestro (Maestro, Schrödinger, LLC, New York, NY, 2016).

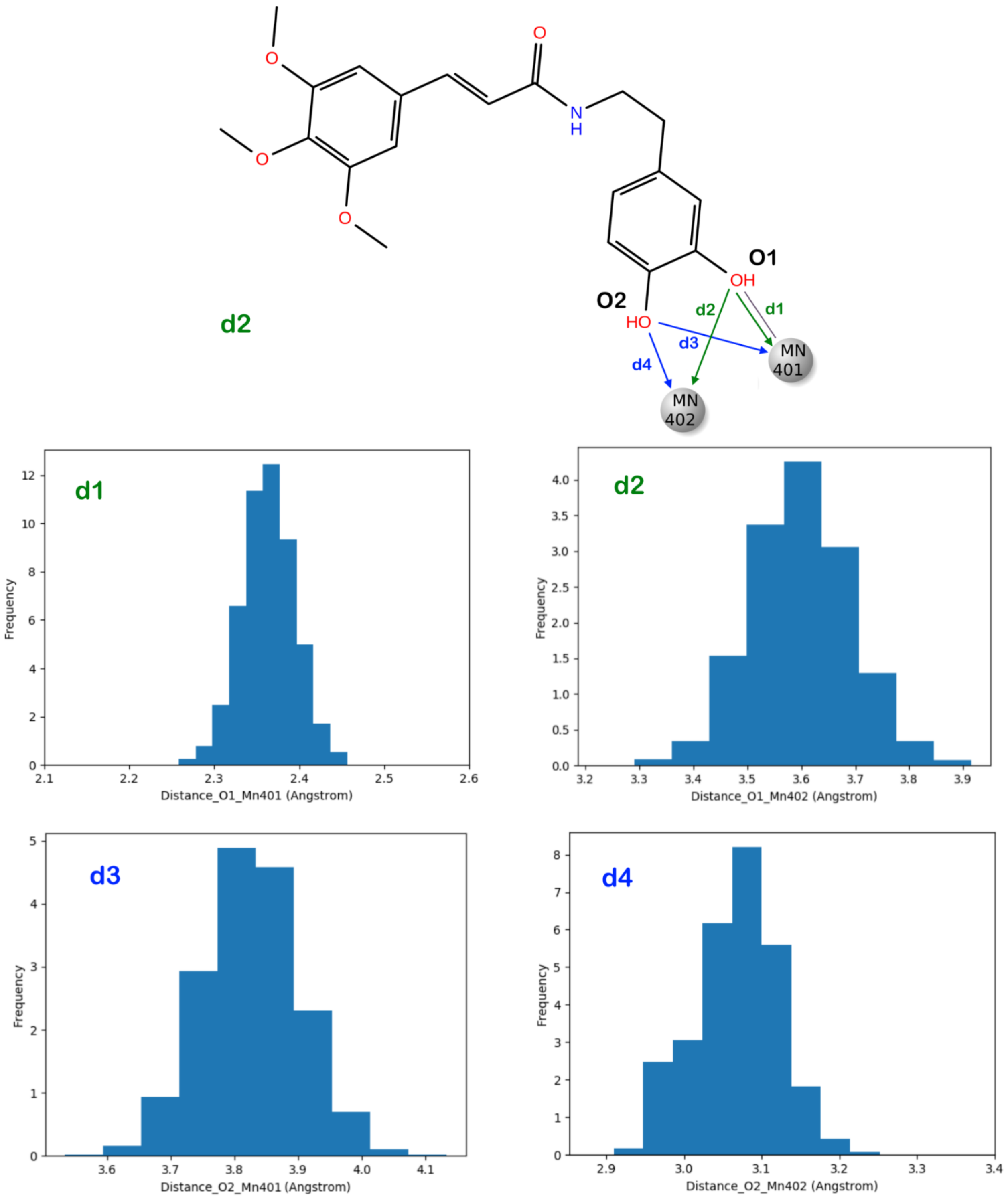

Figure S6. Plots for the selected distances measured during 200 ns of MD simulation of the complex compound **14**/L-ARG.

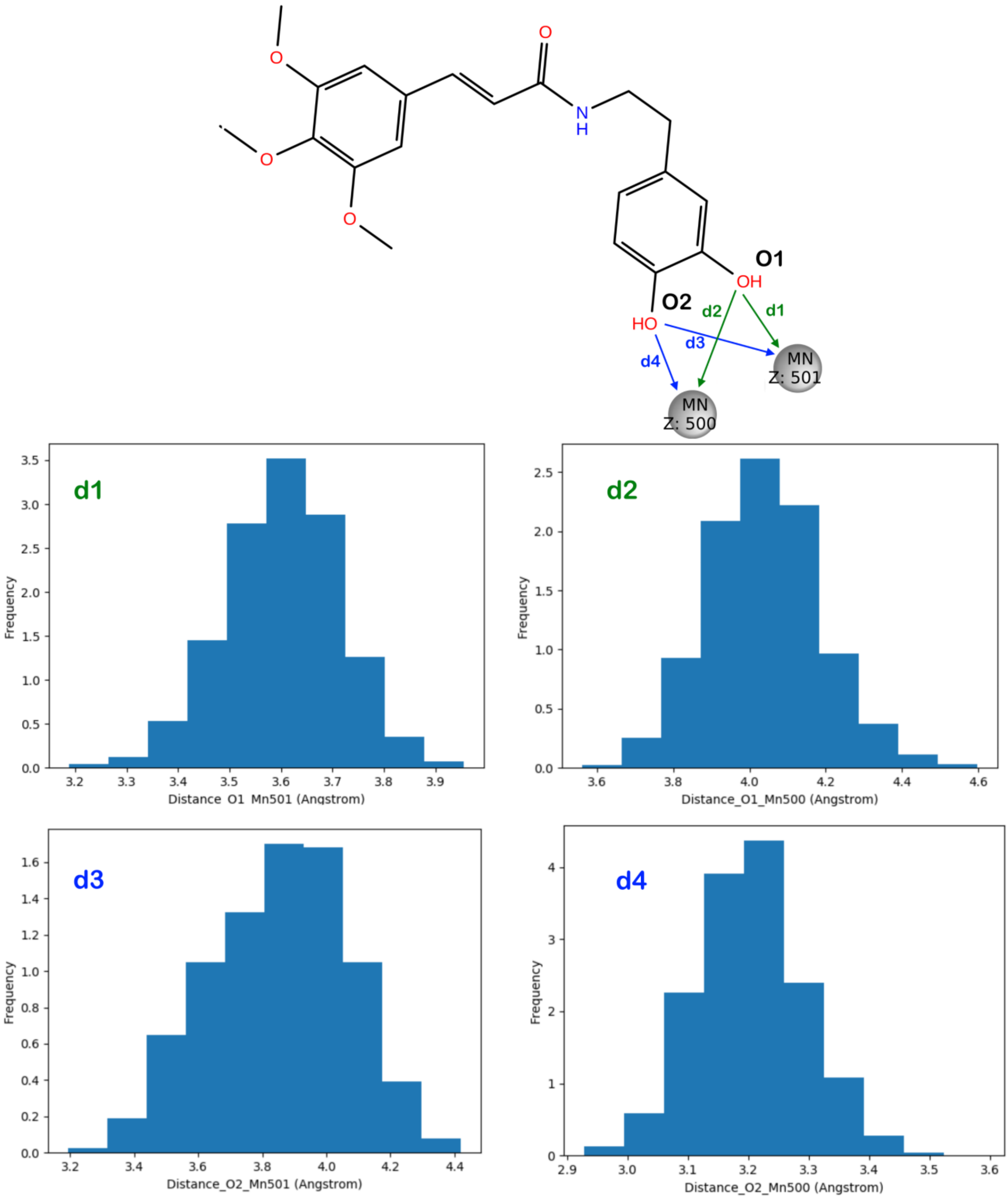

Figure S7. Plots for the selected distances measured during 200 ns of MD simulation of the complex compound **14**/B-ARG.
